# Effects of STN‐DBS surgery on cerebral glucose metabolism and distribution of DAT in Parkinson's disease

**DOI:** 10.1002/brb3.3172

**Published:** 2023-07-17

**Authors:** Ganhua Luo, Xinchong Shi, LuLu Jiang, Lei Wu, Chang Yi, Wenbiao Xian, Yanmei Liu, Fuhua Wen, Hao Qian, Jie Chen, Xiaoli Fu, Jinlong Liu, Xiangsong Zhang, Ling Chen

**Affiliations:** ^1^ Department of Nuclear Medicine The First Affiliated Hospital of Sun Yat‐sen University Guangzhou China; ^2^ Department of Neurology The First Affiliated Hospital of Sun Yat‐sen University Guangzhou China; ^3^ Guangdong Provincial Key Laboratory of Diagnosis and Treatment of Major Neurological Diseases National Key Clinical Department and Key Discipline of Neurology Guangzhou China; ^4^ Department of Neurosurgery The First Affiliated Hospital of Sun Yat‐sen University Guangzhou China

**Keywords:** ^11^C‐CFT, ^18^F‐FDG, dopamine transporter, Parkinson's disease, PET, STN‐DBS

## Abstract

**Introduction:**

: Parkinson's disease (PD) is a neurodegenerative disorder that affects millions of people worldwide. Subthalamic nucleus (STN) deep brain stimulation (DBS) has been shown to be an effective treatment for PD; however, the effects of this surgery on cerebral metabolism and presynaptic dopamine transporter (DAT) distribution are still being studied.

**Methods:**

: In this study, we included 12 PD patients (6 male and 6 female) who underwent STN‐DBS surgery and had both ^18^F‐FDG and ^11^C‐CFT PET/CT imaging before and 1 year after the surgery. We used paired *t*‐tests to identify changes in cerebral metabolism and calculated PD‐related metabolic covariance pattern (PDRP) scores. We also assessed the uptake of ^11^C‐CFT in the striatum using striatal‐to‐occipital ratios (SORs).

**Results:**

: One year after surgery, we observed significant reductions in tremor, rigidity, akinesia, postural instability/gait disturbance, and Unified Parkinson's Disease Rating Scale Part III scores (*p* < .01, *p* < .001, *p* < .001, *p* < .001, and *p* < .001, respectively). Hamilton Depression Rating Scale and quality of life (PDQ‐39 SI) were also significantly reduced (*p* < .05 and *p* < .01, respectively). The mean PDRP score decreased by 37% from 13.0 ± 6.6 to 8.2 ± 7.9 after STN‐DBS surgery (*p* < .05). We observed decreased ^18^F‐FDG uptake in several areas, including the temporal lobe (BA22), thalamus, putamen, and cingulate gyrus (BA24), whereas it was increased in the supplementary motor area, postcentral gyrus, lingual gyrus, and precuneus (*p* < .05). SORs of ^11^C‐CFT in the bilateral caudate nucleus and ipsilateral posterior putamen were significantly decreased compared to preoperative levels (*p* < .05).

**Conclusion:**

: Our findings suggest that STN‐DBS surgery modifies the metabolic network of PD patients and improves motor symptoms, depression, and quality of life. However, it does not prevent the decrease of DAT in striatal areas.

## Graphical abstract

Cerebral glucose metabolism changed one‐year after STN‐DBS surgery. Specifically, regions with reduced metabolism include temporal lobe (BA22), thalamus, putamen and cingulate gyrus (BA24). Meanwhile, the metabolic level of supplementary motor area, postcentral gyrus, lingual gyrus and precuneus increased significantly.

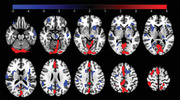

## 1 INTRODUCTION

PD is a progressive neurodegenerative disorder that can lead to metabolic changes in various regions of the central nervous system (Bloem et al., 2021). Parkinson's disease (PD) symptoms encompass the traditional PD triad consisting of tremor, akinesia, and rigidity, which are linked to dysregulation of dopaminergic pathways. Additionally, PD is characterized by various motor symptoms, including gait abnormalities, speech impairments, postural disturbances, and postural instability, which are associated with disruptions in non‐dopaminergic transmission. Furthermore, PD presents a range of non‐motor symptoms, such as cognitive dysfunction, sleep disturbances, reduced sense of smell (hyposmia), depression, and anxiety (Bloem et al., 2021; Isaacs et al., 2020). Although levodopa and other medications can alleviate symptoms for a period of time, drug‐induced dyskinesia and motor fluctuations are common in the late stages of PD for most patients (Evans & Lees, 2004; Lang & Obeso, 2004; Olanow et al., 2006). Subthalamic nucleus deep‐brain stimulation (STN‐DBS) surgery has become widely accepted as an effective method to manage motor symptoms in PD patients (Volonte et al., 2021). However, the mechanism of STN‐DBS remains under investigation.

Numerous past studies have shown that PD patients have specific changes in overall brain metabolism patterns, known as PD‐related metabolic covariance patterns (PDRP) (Meles et al., 2020). These changes are characterized by increased metabolism in the cerebellum, brainstem, and basal ganglia, and decreased metabolism in the motor areas of the parietal and frontal lobes. The score of this metabolic pattern is correlated with the patient's motor symptoms and clinical stage and has been considered an indicator of treatment effectiveness in some studies. In addition, some studies have explored metabolic networks associated with PD patients’ motor/cognitive function through network analysis, such as the PD‐related cognitive network (PDCP) and the PD‐related tremor network (PDTP) (Mure et al., 2011; Schindlbeck et al., 2021). These metabolic patterns can be used to study the mechanisms of specific symptoms and monitor relevant treatment effects.

Molecular imaging is an important tool in clinical research for patients with PD (Haider et al., 2023). Dopamine synthesized in the substantia nigra neurons needs to be transported to the presynaptic membrane through monoamine vesicles and released into the synaptic cleft, and then reuptaken by the presynaptic membrane's dopamine transporter (DAT). By using molecular probes to track the distribution of biomolecules such as DAT and VMAT2, the damaged condition of substantia nigra neurons in patients can be visually displayed, and it can be used for semiquantitative analysis to detect disease progression and assess treatment efficacy. Animal experiments have also shown a strong correlation between the distribution of DAT obtained from PET imaging and the number of surviving substantia nigra neurons (German et al., 2015).

The purpose of this study is to investigate the effects of STN‐DBS on the distribution of presynaptic DAT and the pattern of cerebral glucose metabolism using ^11^C‐CFT and ^18^F‐FDG PET imaging in PD patients before and after surgery.

## 2 METHODS

### 2.1 Study subjects

We consecutively recruited 12 patients with PD from 2011 to 2017 (mean age 61.3 ± 6.1 years, 6 males and 6 females) who were diagnosed with idiopathic PD and asked for STN‐DBS surgery. Inclusion and exclusion criteria for participants in this study are presented in the following table.

**Table:** 

**Inclusion criteria**	**Exclusion criteria**
Diagnosis of idiopathic PD according to the British PD Association Brain Bank criteria	Presence of cognitive impairment (mini‐mental state examination [MMSE] score <26)
Age: 18–75 years	Severe psychiatric or behavioral disorders
Disease duration: more than 5 years	Severe metabolic, cardiac, respiratory, renal, and hepatic conditions
Severe levodopa‐induced motor complications despite optimal adjustment of antiParkinsonian drugs	Inability to comply with the study protocol
At least 30% improvement in motor symptoms assessed by Unified Parkinson's Disease Rating Scale Part III (UPDRS‐III) after a levodopa challenge test	
Normal brain magnetic resonance imaging (MRI)	

The study was approved by the Medical Ethics Committee of the First Affiliated Hospital of Sun Yat‐sen University, and a signed informed consent form was obtained from each participant. All patients underwent both ^18^F‐FDG and ^11^C‐CFT PET/CT imaging preoperatively and 1 year after STN‐DBS surgery with stimulation on.

### 2.2 Clinical profile

In this study, clinical assessment was conducted during the “off” phase, with patients abstaining from anti‐PD medication for more than 12 h. The assessment was performed by movement disorders specialists who are neurologists. Clinical motor symptoms were evaluated using UPDRS‐III. Sleep disruption was evaluated using the PD sleep scale (PDSS). The severity of depression and anxiety was assessed using the Hamilton Depression Rating Scale (HAMD) and the Hamilton Anxiety Scale (HAMA), respectively. Cognitive impairment was assessed using the MMSE and the Montreal Cognitive Assessment (MoCA). In addition, quality of life was assessed using the PDQ‐39 SI, which ranges from 0 (no impairment) to 100 (maximum impairment).

The implantable pulse generator (IPG) was activated 1 month after surgery. Patients were asked to return to the clinic for follow‐up 1 year after STN‐DBS surgery with stimulation on. Doses of antiParkinsonian medication were recorded at each follow‐up visit. Medication was calculated as the total levodopa equivalent daily dose (LEDD) for each patient. Medications were converted to an equivalent dose of immediate‐release levodopa (Madopar, Roche) using the following formula: 100 mg immediate‐release levodopa = 133 mg extended‐release levodopa = 1 mg pramipexole = 100 mg piribedil = 10 mg selegiline. The efficacy of levodopa was increased by 25% compared with entacapone (Jiang et al., 2015).

### 2.3 STN‐DBS surgery

On the day prior to surgery, all patients underwent a brain MRI scan. A Leksell G‐frame (Elekta AB) was secured to the skull during the preoperative brain CT scan. Subsequently, the CT image was merged with the MRI image for each patient using stereotactic planning software (iPlan, Brainlab) to determine the target and plan the trajectory. Electrode implantation was performed using stereotactic guidance and microelectrode recording (MER) technique under local anesthesia. The quadripolar leads (model 3389, Medtronic) were inserted at the target positions once satisfactory signals were obtained from MER. Intraoperative experimental stimulation was performed to evaluate the improvement in PD symptoms and stimulation‐related side effects. After confirming accurate placement of the electrode, the lead was secured with a fixation device (Stimloc, Medtronic) to the drilled site. The implantation of leads on the opposite side was conducted using the same procedure. Finally, an IPG (Kinetra, Medtronic) was subcutaneously implanted in the right subclavicular area and connected to the extended leads under general anesthesia (Jiang et al., 2015).

### 2.4 ^18^F‐FDG PET/CT imaging

^18^F was produced using a Cyclone‐10 cyclotron (IBA) at our PET center, and the synthesis of ^18^F‐FDG was performed using a fully automated synthesizer (Synthera, IBA). Subsequently, ^18^F‐FDG PET/CT imaging was conducted on a Gemini GXL16 (Philips). Prior to imaging, patients were required to fast for at least 6 h and be in the “off” period. After the injection of 370–444 MBq ^18^F‐FDG, patients rested for 45 min and underwent a low‐dose CT scan of the brain for attenuation correction of the PET images. PET images were acquired for 10 min in 3D mode with an axial field of view of 180 mm. The image was reconstructed using the 3D Ramla algorithm to obtain a PET image of the brain with a resolution of 2 mm × 2 mm × mm (Xian et al., 2021).

### 2.5 ^11^C‐CFT PET/CT imaging

At our center, an automated method was developed for the synthesis of ^11^C‐CFT tracer. Cyclone‐10 cyclotron‐produced ^11^C‐CO2 was used as the starting material, which was first hydrolyzed with lithium aluminum hydride (LiAlH) to form a salt, followed by hydrolysis with hydrogen iodide to produce ^11^C‐CH3I. Subsequently, ^11^C‐CH3I was converted to triflate‐^11^C‐CH3 using an Ag triflate/C column. Finally, ^11^C‐CFT was synthesized by methylation with the precursor nor‐β‐CFT, eluted with anhydrous ethanol, and filtered through a sterile membrane. The putative purity was determined by high‐performance liquid chromatography to be >95%. All PD patients were in the “off” phase prior to PET imaging. Patients were injected with ^11^C‐CFT (average 185–370 MBq) and rested for 1 h before imaging. The imaging procedure was the same as that used for ^18^F‐FDG.

### 2.6 ^18^F‐FDG image processing

The DICOM data were converted to the SPM analysis format using MRIConvert software (v2.1.0). Subsequently, the ^18^F‐FDG images were normalized to the standard Montreal Neurological Institute (MNI) brain spatial coordinate system using SPM. The spatially normalized PET images were then smoothed with an 8 mm isotropic Gaussian kernel to account for interindividual anatomical variability. Finally, paired *t*‐test analysis was conducted to identify the regions with metabolic changes, and PDRP scores were calculated using SSM/PCA.

### 2.7 ^11^C‐CFT image processing

The T1‐weighted images from the MRI and the PET images (pre‐ and postoperatively) were first aligned. The MRI images were subsequently normalized to the MNI coordinate system, and the PET images were normalized using the same transformation parameters. Regions of interest in the striatum and occipital lobe were extracted from the normalized MRI images. Each side of the striatum was further divided into the head of the caudate nucleus, the body of the caudate nucleus, the tail of the caudate nucleus, the anterior part of the putamen, and the posterior part of the putamen. Finally, the mean radioactivity counts in the striatum and occipital cortex were used to calculate the striatal‐to‐occipital ratios (SORs) of ^11^C‐CFT (Jokinen et al., 2009). As PD patients typically have a lateralized onset, they were uniformly compared and analyzed according to the contralateral and ipsilateral sides of onset.

### 2.8 Statistical analysis

SPSS 26 software was used for statistical analysis in this study. The Shapiro–Wilk test was utilized to assess the normality of the distribution of the clinical scores. When parametric tests were applicable, clinical scores from baseline to follow‐up were tested using Wilcoxon signed‐rank or paired *t*‐tests. Metabolic changes were analyzed by paired *t*‐tests based on voxel levels using SPM software, and FDR‐corrected *p* < .05 was considered statistically significant. The xjView toolbox (https://www.alivelearn.net/xjview) was used to display the metabolism of each brain region. Paired *t*‐tests were performed for SORs of ^11^C‐CFT in each striatal region. Spearman correlation analysis was conducted between preoperative PDRP and preoperative UPDRS‐III scores. A *p*‐value of <.05 was considered statistically significant.

## 3 RESULT

### 3.1 Clinical assessment

Twelve PD patients (6 male and 6 female) were included in this study, with a mean age of 61.3 ± 6.1 years, mean disease duration of 11.3 ± 5.1 years, and mean UPDRS‐III‐OFF score of 54.7 ± 13.1 (Tables 1 and 2). All patients underwent STN‐DBS surgery and no cases of persistent neurological sequelae or electrode dislocation after surgery were reported. One year after STN‐DBS, there was a reduction in tremor, rigidity, akinesia, PI/GD, and UPDRS‐III scores, with a 49% reduction in tremor (*p* < .01), 58% reduction in rigidity (*p* < .001), 37% reduction in akinesia (*p* < .001), 33% reduction in PI/GD (*p* < .001), 42% reduction in UPDRS‐III scores (*p* < .001), and a 21% reduction in LEDD (*p* = .15). The HAMD and PDQ‐39 SI scores were also reduced by 31% (*p* < .05) and 37% (*p* < .01), respectively. However, there were no significant changes in the PDSS, HAMA, MMSE, and MoCA scores at 1 year after STN‐DBS.

**TABLE 1:** Clinical profiles of all patients.

**Age (years)**	61.3 ± 6.1
**Gender (M/F)**	6/6
**Disease duration (years)**	11.3 ± 5.1
**Hoehn and Yahr stages (III/IV/V)**	9/2/1

**TABLE 2:** Clinical assessment of patients.

A. Motor symptoms and LEDD (mean ± SD)						
	Tremor	Rigidity	Akinesia	PI/GD	UPDRS‐III	LEDD (mg)
**Baseline (*n* = 12)**	9.9 ± 7.2	11.7 ± 1.6	22.5 ± 5.1	8.1 ± 3.1	54.7 ± 13.1	970.3 ± 523.9
**1 year (*n* = 12)**	5.1 ± 4.2	4.9 ± 3.8	14.2 ± 5.7	5.5 ± 3.1	31.9 ± 12.5	763.0 ± 492.5
**1 year vs. baseline**	**	***	***	***	***	*p* = .15

**B. Nonmotor symptoms (mean ± SD)**						
	**PDSS**	**HAMD**	**HAMA**	**MMSE**	**MoCA**	**PDQ‐39 SI**
**Baseline (*n* = 12)**	101.7 ± 18.6	11.7 ± 6.3	7.1 ± 4.4	27.0 ± 4.0	23.2 ± 5.7	33.0 ± 10.3
**1 year (*n* = 12)**	110.6 ± 21.3	8.1 ± 3.9	4.6 ± 2.2	27.8 ± 1.8	24.0 ± 4.4	20.9 ± 14.1
**1 year vs. baseline**	*p* = .13	*	*p* = .96	*p* = .36	*p* = .35	**

Abbreviations: HAMA, Hamilton Anxiety Scale; HAMD, Hamilton Depression Scale; LEDD, levodopa equivalent daily dose; MMSE, mini‐mental state examination; MoCA, Montreal Cognitive Assessment; PDQ‐39 SI, the 39‐item Parkinson's disease questionnaire summary index score; PDSS, Parkinson's disease sleep scale; PI/GD, posture instability/gait disturbance; UPDRS, Unified Parkinson Disease Rating Scale; UPDRS‐III, Unified Parkinson's Disease Rating Scale Part III.

*p < .05.

**p < .01.

***p < .001.

### 3.2 ^18^F‐FDG metabolism

**FIGURE 1:**
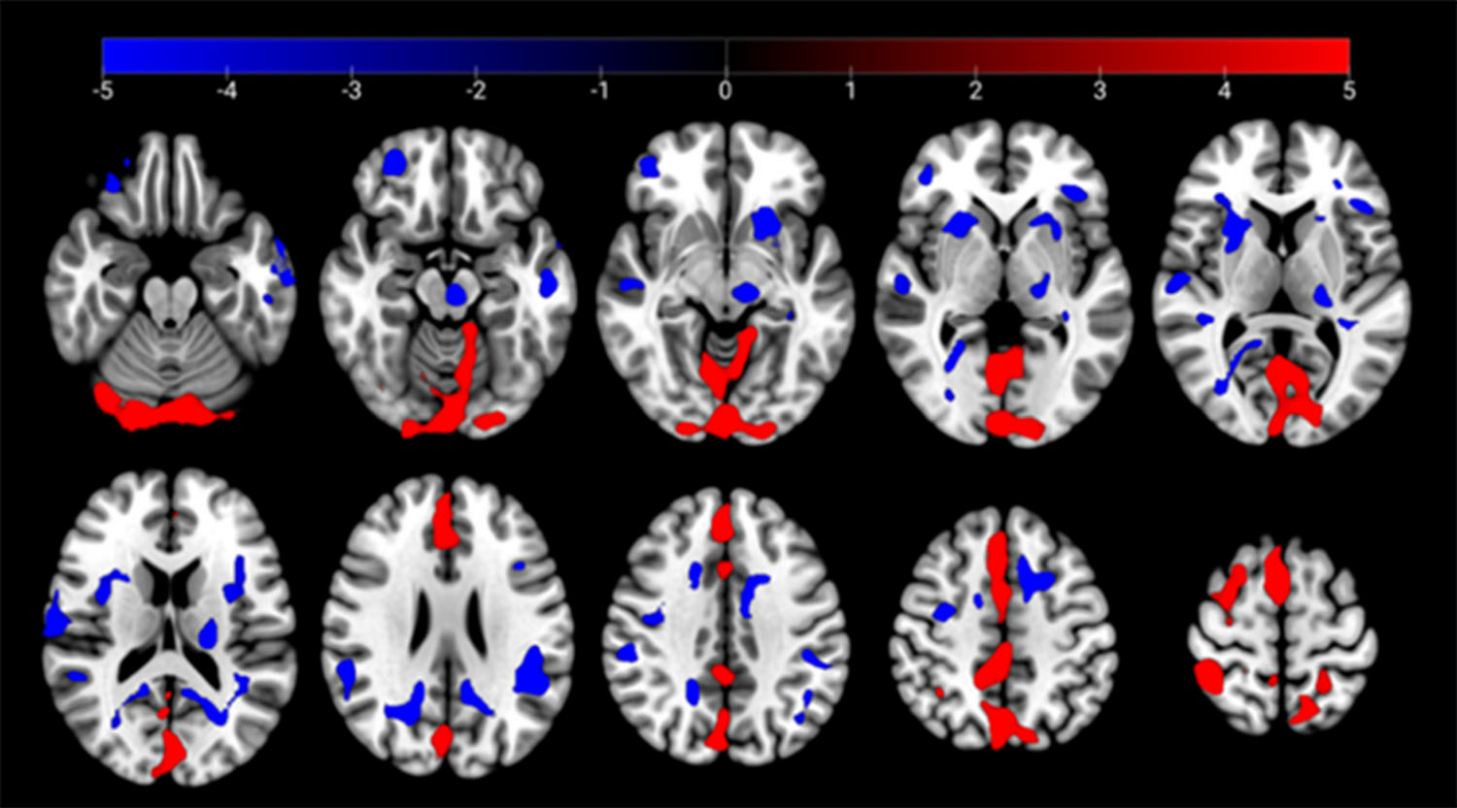
^18^F‐FDG metabolic changes 1 year after the subthalamic nucleus‐deep brain stimulation (STN‐DBS) surgery (*p* < .01, FDR < .05). “Red” indicates the regions with increased metabolism, “blue” indicates the regions with decreased metabolism (cluster size >50 voxels).

After surgery, there was a significant decrease in PDRP scores by 37% with a change from 13.0 ± 6.6 to 8.2 ± 7.9 (*p* < .05). Additionally, preoperative PDRP scores demonstrated a positive correlation with preoperative UPDRS‐III scores (*r* = .63, *p* < .05). The study also found a significant change in regional cerebral metabolism after surgery, with areas such as the supplementary motor area (SMA), postcentral gyrus, lingual gyrus, and precuneus showing increased ^18^F‐FDG uptake. Conversely, regions such as the temporal lobes (BA 22), thalamus, putamen, and cingulate gyrus (BA 24) showed reduced ^18^F‐FDG uptake (cluster size >50 voxels, *p* < .05) (Table 3 and Figure 1).

**TABLE 3:** Brain regions with significant ^18^F‐FDG metabolic changes after subthalamic nucleus‐deep brain stimulation (STN‐DBS) surgery.

Item	Brain region	MNI coordinates			*Z*_max_	Cluster size (voxels)
		*X*	*Y*	*Z*		
**Metabolic decreases**	Cingulum_Mid_L(BA24)	−12	4	40	−5.1	291
	Temporal_Sup_R(BA22)	52	−14	8	−4.2	80
	Thalamus_L	−4	−22	−6	−4.2	85
	SupraMarginal	−44	−54	32	−4.1	347
	Putamen	−28	12	−4	−4.1	211
**Metabolic increases**	Lingual_R	4	−62	2	5.5	536
	Cerebelum_Crus1_R	16	−90	−26	5.1	1012
	Precuneus	12	−40	44	4.4	279
	Supp_Motor_Area	6	14	62	4.0	546
	Postcentral	20	−40	60	4.0	511

Abbreviation: MNI, Montreal Neurological Institute.

### 3.3 ^11^C‐CFT distribution

**FIGURE 2:**
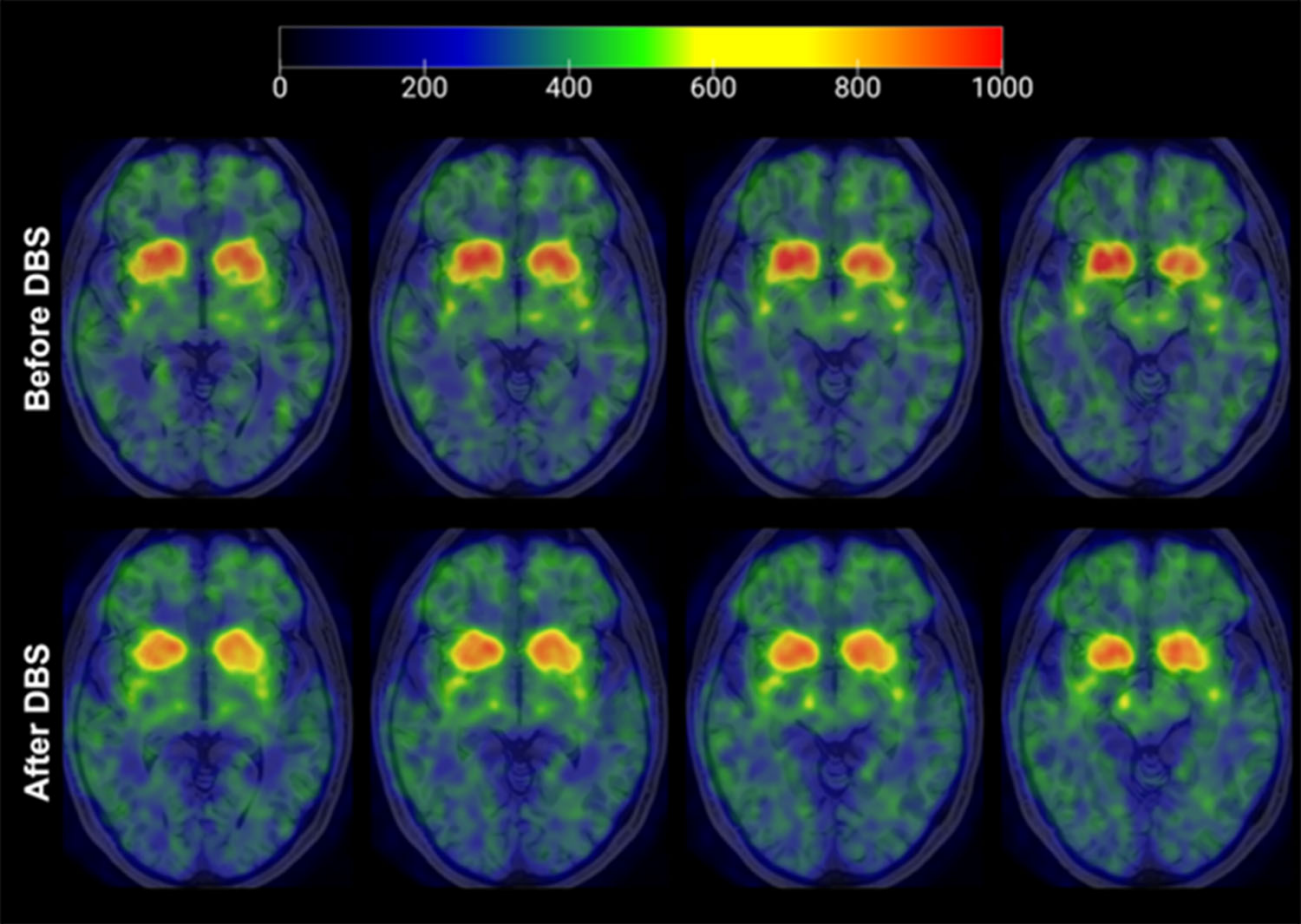
^11^C‐CFT imaging results of a 60‐year‐old male patient before (a) and after (b) subthalamic nucleus‐deep brain stimulation (STN‐DBS) surgery. Striatal dopamine transporter (DAT) distribution is reduced 1 year after STN‐DBS.

As depicted in Table 4, there were no significant differences in SORs of ^11^C‐CFT between the contralateral and ipsilateral anterior putamen. However, SORs of the bilateral caudate nucleus and ipsilateral posterior putamen showed significant changes after surgery (*p* < .05). Specifically, there was a 3.6% decline in SORs in the contralateral posterior putamen, a 4.8% decline in the contralateral caudate nucleus, and a 7.3% decline in the ipsilateral caudate nucleus. These findings suggest that STN‐DBS surgery does not prevent the decrease of DAT distribution in the striatal region (Figure 2).

**TABLE 4:** Striatal dopamine transporter (DAT) striatal‐to‐occipital ratios (SORs) before and after subthalamic nucleus‐deep brain stimulation (STN‐DBS) (mean ± SD).

		APu	PPu	CNH	CNB	CNT
**Ipsilateral**	**Before**	1.61 ± 0.15	1.37 ± 0.08	1.84 ± 0.26	1.60 ± 0.20	1.44 ± 0.29
	**After**	1.59 ± 0.16	1.32 ± 0.07	1.76 ± 0.24	1.53 ± 0.19	1.36 ± 0.25
	***p***	ns	*	*	*	**
**Contralateral**	**Before**	1.73 ± 0.17	1.40 ± 0.07	1.95 ± 0.25	1.73 ± 0.22	1.42 ± 0.17
	**After**	1.70 ± 0.16	1.38 ± 0.06	1.82 ± 0.25	1.60 ± 0.21	1.31 ± 0.17
	***p***	ns	ns	*	**	**

Abbreviations: APu, anterior putamen; CN, caudate nucleus; CNB, caudate nucleus body; CNH, caudate nucleus head; CNT, caudate nucleus tail; ns, no significant; PPu, posterior putamen; Pu, putamen.

*p < .05.

**p < .01.

## 4 DISCUSSION

In this study, we investigated the impact of STN‐DBS surgery on striatal DAT distribution and cerebral metabolism. Our findings indicated that STN‐DBS could modify the cerebral network without preventing striatal DAT decline. On the other hand, UPDRS‐III scores, particularly resting tremor and rigidity, were significantly reduced after STN‐DBS surgery, confirming that STN‐DBS is an effective therapeutic approach in controlling symptoms in patients with PD.

Our study found that preoperative PDRP was correlated with preoperative UPDRS‐III scores (Ge et al., 2020). Changes in brain glucose metabolism were noted in several regions, with decreased metabolism in cingulate, superior temporal, putamen, and thalamus, and increased metabolism in lingual, precuneus, postcentral gyrus, and SMA. These findings were in line with previous studies (Ge et al., 2020; Langner‐Lemercier et al., 2015). Kotaro's research revealed that bilateral STN‐DBS decreased glucose metabolism in specific brain regions, including the putamen, globus pallidus, primary motor area, prefrontal cortex, and cerebellar vermis (Asanuma et al., 2006). The manifestations of akinesia and rigidity in PD are believed to be associated with abnormal functions of the cortico‐striatopallido‐thalamocortical motor circuits, whereas tremor is associated with abnormalities of the cerebello‐thalamo‐cortical pathway (Mure et al., 2011). The improvement of these symptoms may be attributed to the modulation of these pathways, which can also be confirmed through neuroimaging studies of cerebral metabolism (Poston & Eidelberg, 2012).

In our study, the DAT distribution was consistent with previous studies showing that dopamine function remained persistently decreased in the caudate nucleus and ipsilateral posterior putamen, indicating that STN‐DBS did not prevent nigrostriatal neuronal apoptosis. However, the lower rate of decline per year compared to previous studies may be due to differences in disease progression or receptor target selection. It is also worth noting that the results of DAT distribution in the ipsilateral anterior putamen and the contralateral putamen after STN‐DBS surgery were not statistically different in our study, possibly due to the late stage of the patients or the limited resolution of PET. Jacobs suggests that STN‐DBS has complex electrical effects on individual neurons and neuronal networks, which can alter the dynamics of neurotransmitters, shape the microenvironment, and influence neuroplasticity, neuroprotection, and neurogenesis (Jakobs et al., 2019; Miller et al., 2021). However, several studies have found that there was no significant change in striatal dopamine concentration after surgery, and that STN‐DBS did not have a neuroprotective effect on STN targets (Hilker et al., 2003, 2005).

Research on STN‐DBS has demonstrated positive effects on various NMS in patients with PD, such as sweating, sleep/fatigue, mood/cognition, attention/memory, perceptual/hallucinations, urinary symptoms, pain, and olfactory function (Cury et al., 2014; Dafsari et al., 2018, 2020; Jost et al., 2020). In our study, we utilized the PDSS, HAMD, HAMA, MMSE, MoCA, and PDQ‐39 SI to assess sleep, depression, anxiety, cognitive impairment, and quality of life. While sleep did not significantly improve 1 year after STN‐DBS, we observed significant improvements in depression which is consistent with previous research that suggests STN‐DBS may regulate the limbic system and lead to improvements in depression (Cartmill et al., 2021). The lingual gyrus is believed to play a role in episodic memory consolidation. The activity of the lingual gyrus is associated with happiness and loneliness, suggesting that the lingual gyrus is involved in emotional processes (Kong et al., 2015). PD patients with depression may benefit from lingual gyrus glucose metabolism increasing after STN‐DBS. Future research is needed to better understand the underlying mechanisms of these improvements and to identify patients who are most likely to benefit from STN‐DBS for non‐motor symptoms.

Reducing the LEDD can have significant benefits in reducing medication‐related dyskinesias, motor fluctuations, and other side effects, which is a primary aim of STN‐DBS surgery (Nakajima et al., 2018). Research has shown that Parkinson's patients who undergo STN‐DBS surgery require significantly less levodopa following the procedure (Krack et al., 2003). It is generally recommended to use the lowest possible drug dosage to achieve satisfactory symptom control, which can help reduce motor complications and allow for future medication adjustments. Our research found that at 1‐year follow‐up after surgery, the patients’ LEDD decreased by 21%. However, the paired test comparison before and after surgery did not have statistical significance (*p* = .15). This is different from other research results, mainly because the two advanced patients included in the study had an increase in LEDD after surgery, whereas the LEDD of the other 10 patients decreased by an average of 36% (*p* = .02). This also indicates that the surgical outcome of STN‐DBS still has individual differences.

This study had some limitations that need to be taken into consideration. First, there were no nonsurgical controls at the same stage of the disease, which limits our ability to conclude whether STN‐DBS slowed the degeneration of substantia nigra cells. Secondly, the sample size was relatively small, and thus the results of the study need to be verified further to ensure their accuracy and generalizability. Despite these limitations, the study provides valuable insights into the effects of STN‐DBS on DAT density in PD patients and adds to the existing body of literature on the topic.

## 5 CONCLUSION

In conclusion, STN‐DBS surgery did not prevent the decrease of striatal DAT density, but it modified the cerebral metabolism.

## AUTHOR CONTRIBUTIONS

Research project: Ling Chen and Xiangsong Zhang designed and organized this study. Jinlong Liu reviewed the study design and gave valuable suggestions.

Research execution and statistical analysis: Lulu Jiang, Hao Qian, Jie Chen, Xiaoli Fu, Lei Wu, Wenbiao Xian, and Yanmei Liu selected the PD patients, assessed their motor symptoms, prepared them for STN‐DBS surgery and made the follow‐up. Jinlong Liu performed the STN‐DBS surgery. Fuhua Wen prepared the radiotracers ^18^F‐FDG and ^11^C‐CFT. Xinchong Shi, Ganhua Luo, and Chang Yi were responsible for PET/CT imaging and data analysis.

Manuscript writing: Finally, Ganhua Luo, Xinchong Shi, and Lulu Jiang completed the initial writing, and then Ling Chen and Xiangsong Zhang reviewed and revised the manuscript.

## CONFLICT OF INTEREST STATEMENT

The authors declare that they have no conflicts of interest.

### PEER REVIEW

The peer review history for this article is available at https://publons.com/publon/10.1002/brb3.3172.

## CONSENT TO PUBLICATION

Consent for publication had been obtained from the subjects whose imaging results were displayed in this article.

## Data Availability

The data that support the findings of this study are available on request from the corresponding author. The data are not publicly available due to privacy or ethical restrictions.
